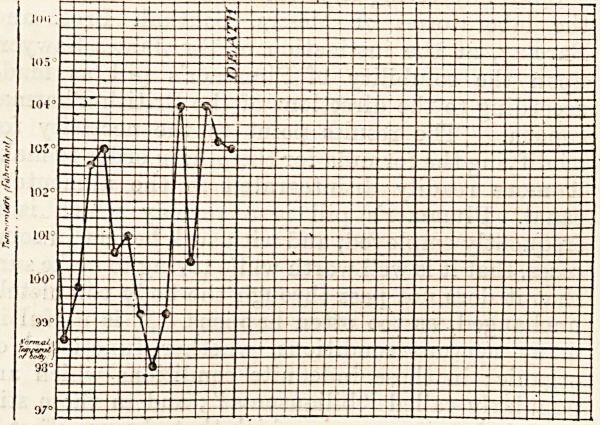# Pyrexia in Uncomplicated Cirrhosis of the Liver

**Published:** 1907-05-25

**Authors:** 


### Clinical Points.

### PYREXIA IN UNCOMPLICATED CIRRHOSIS OF THE LIVER.

Any patient who has cirrhosis of the liver is liable to intercurrent inflammatory troubles which may cause pyrexia. There may be acute pleurisy, for example; or phthisis, lobar pneumonia, general tuberculosis, an abscess or abscesses in the subcutaneous tissues or elsewhere, and so on. It is not of cases in which there is an additional and often obvious cause for pyrexia that we are speaking just now, but of cases in which the cirrhosis is proved by autopsy to have been the only gross pathological change in the body.

Carrington believed that if the temperature were recorded throughout the disease, there would be few patients with uncomplicated cirrhosis of the liver who would not at one time or another exhibit pyrexia. In the early stages microscopical sections of the liver show widespread infiltration of the portal areas with small round cells; even in the later stages the cirrhotic changes indicate a persistently recurring inflammation of low type. If this be so, it would almost be expected that at least during the exacerbations of the intrahepatic inflammation there would be pyrexia. It is of considerable clinical value to know that moderate pyrexia is not only compatible with a diagnosis of uncomplicated cirrhosis of the liver, but sometimes even helps to confirm it. In alcoholic subjects pyrexial attacks which seem to have no cause are by no means uncommon; in such cases the possibility of hepatic cirrhosis must not be overlooked.

The pyrexia in typical cases is not extreme; the temperature may be about 100° F. every evening, and 98° F. every morning; as a rule the rise is to about the same height night after night, whilst there is seldom any drop far below normal in the morning. Sometimes, however, the pyrexia may be less regular, may reach much higher levels than 100° F., and need not always return to normal in the morning. Occasionally, so high a figure as 104° F. may be touched, without any rigor. The following is an example of one of these extreme pyrexial cases:—

A female patient, aged 42, came under observation when she had been out of health for four months. She was a married woman, with a good family history, and had suffered from no previous illness of importance. She had had one miscarriage, but no children. Her occupation was house-work. She had been in the habit of drinking ale in moderate quantity, but said that she never took spirits. Four months before she was seen she began to feel weak, to vomit constantly after her meals, to suffer from profuse diarrhœa, and at night from burning sensations in the soles of her feet, and to perspire a great deal. She began to lose flesh rapidly. The vomiting gradually increased in severity, particularly before breakfast in the morning, though she was also liable to it throughout the day after meals. She never suffered from hæmatemesis, and there was no abdominal swelling nor pain, and little nausea. The diarrhœa increased to the extent of from five to seven evacuations of liquid and offensive stools a day. There was slight œdema of the ankles and feet after she had been standing for an hour or so after getting up in the morning. She was anæmic, with a slightly yellow tinge in the skin, but without any jaundice of the conjunctivæ, and without bile pigments in the urine. She looked worn and ill, and by the time she came under observation she was glad to remain in bed, where she rested comfortably at full length. The liver was felt, firm but smooth, two inches below the costal margin, and it was slightly tender. The spleen was not felt. The cardiac and pulmonary physical signs were normal except that there was evidence of emphysema. There was a slight cough but no expectoration. The respirations were 29 per minute, and the pulse, which was small and weak, had a rate of 110 per minute. The urine was high-coloured, but had a specific gravity of only 1010; it contained neither albumen, sugar, nor bile pigment. The patient was irritable, but her mental faculties were good; there was no definite evidence of any peripheral neuritis, unless the pains in the soles of the feet at night were due to this; the knee-jerks were apparently normal. The tongue and fauces were dry; the former was covered by a slight white fur, and there were extensive sordes on the lips and teeth. The diagnosis was by no means obvious, but among other things cirrhosis of the liver was suspected, and this was confirmed later by autopsy. From our present point of view the pyrexia was the main thing to attract attention; the patient was seen daily for four weeks before her death, and the illustration given is her temperature chart.

Before she died her liver increased in size, reaching nearly to the umbilicus towards the end. The patient herself changed but little, except that she gradually declined; three days before her death the vomiting became much worse, the pulse-rate rose to 124, and she became semi-comatose. At this time the urine was very scanty and high coloured, with a specific gravity of 1020; it was still free from albumen, blood, sugar, and bile, but contained very large quantities of indican. Complete coma set in upon the last day, and in this coma she died. The duration of the illness from the day she began to complain until the day she died was exactly five months.

At the autopsy the brain and its meninges, the pleuræ, pericardium, pancreas, suprarenal capsules, and kidneys were natural; the lungs were emphysematous in the upper lobes and œdematous in the lower, but free from tubercle and from pneumonia; the heart muscle was flabby; and the stomach and intestines were congested and coated internally with mucus. The main pathological changes were in the spleen and liver. The former, though not felt during life, was moderately enlarged (8 ounces), as is almost the rule in cirrhosis of the liver. The latter was very large, hard, and smooth, weighing 72 ounces, tough, and of a pale yellow colour. It was a typical example of a unilobular cirrhotic liver, and in microscopical sections there was an enormous degree of nuclear proliferation of the portal connective tissue cells, and much fatty change in the cells of the liver substance, which probably accounts for the patient dying of cholæmia in this early stage of the disease. If the patient had recovered for the time being it is very probable that the diagnosis of the cause of the pyrexia would have remained obscure; similar cases in alcoholic subjects are constantly coming before one; we must remember that a possible, and not uncommon, cause for such pyrexia is cirrhosis of the liver, and that with cirrhosis of the liver pyrexia is likely to occur at some stage of the disease, even in the absence of any complication.

## Figures and Tables

**Figure f1:**
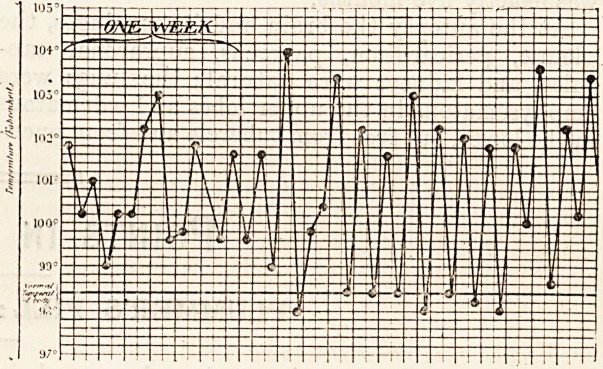


**Figure f2:**